# Interaction between DEAD-box RNA helicase 10 and influenza PB1 polymerase selectively regulates influenza A virus replication

**DOI:** 10.1128/jvi.00815-26

**Published:** 2026-09-22

**Authors:** Rizwan Ullah, Wei Chen, Lingkai Zhang, Chaoxuan Guo, Bin Ren, Xinyi Pei, Dong Yang, Qingshuai Sun, Xinyu Gao, Hongbo Zhou, Liurong Fang, Wen Su

**Affiliations:** 1 National Key Laboratory of Agricultural Microbiology, College of Veterinary Medicine, Huazhong Agricultural University 47895, Wuhan, Hubei, China; 2 Hubei Hongshan Laboratory, Wuhan, Hubei, China; 3 Frontiers Science Center for Animal Breeding and Sustainable Production, Wuhan, Hubei, China; 4 The Cooperative Innovation Center for Sustainable Pig Production, Huazhong Agricultural University 47895, Wuhan, China; 5 Shennongjia Science and Technology Innovation Center, Shennongjia Multidisciplinary Observation and Research Station of the Ministry of Agriculture and Rural Affairs, Huazhong Agricultural University 47895, Wuhan, Hubei, China; Emory University School of Medicine, Atlanta, Georgia, USA

**Keywords:** influenza A virus, DDX10, virus replication, polymerase activity, strain specificity, cross-species infection, PB1

## Abstract

Identifying the host factors that mediate avian influenza virus adaptation in mammals is important for monitoring zoonotic potential. Although viral polymerase adaptations are known to influence cross-species transmission, the engagement of specific host factors with divergent viral polymerases remains to be explored. We examined human DEAD-box RNA helicases (DDXs) as potential regulators of influenza polymerase activity. A screen of 16 DDXs identified DDX10 as a factor that selectively enhanced the polymerase activity and replication of human-adapted H1N1 viruses, including the 2009 pandemic strain, but not avian-origin H9N2 viruses. This differential activity was associated with DDX10 showing stronger interaction with human-origin PB1 (Cal04/H1N1) than with avian-origin PB1 (BJ16/H9N2). Strain specificity was mediated by two residues (336 and 364) within the PB1 catalytic domain: introducing H1N1-type residues (I336 and I364) into H9N2-PB1 conferred DDX10 responsiveness, while reciprocal mutations in H1N1-PB1 abolished it. This work defines DDX10 as a potential host factor that differentially supports influenza polymerase activity, revealing a specific molecular interface that contributes to the replication efficiency of distinct viral subtypes in human cells and providing insight into host-adaptive mechanisms.

**IMPORTANCE:**

This study identifies DDX10 as a strain-specific host factor that differentially regulates influenza A virus replication. By demonstrating that DDX10 selectively enhances human-adapted H1N1 viruses over avian-origin H9N2 viruses through direct interaction with the viral PB1 protein, and by mapping two critical residues (positions 336 and 364) in PB1 that govern this selectivity, our work provides a mechanistic framework for understanding how a single host factor can modulate viral fitness and host adaptation. These findings have important implications for predicting cross-species transmission potential and may guide the development of host-targeted antiviral strategies.

## INTRODUCTION

Influenza A viruses (IAVs) remain a significant threat to public health, causing both seasonal epidemics and sporadic pandemics (1–4). A key factor determining viral replication efficiency and host adaptation is the viral RNA-dependent RNA polymerase (RdRp), composed of the PB2, PB1, and PA subunits. Together with the nucleoprotein (NP), the RdRp forms viral ribonucleoprotein (vRNP) complexes that carry out viral transcription and replication (5–7). Successful adaptation of this replication machinery to host cellular factors is a critical step for viral emergence in new species (8–10).

Host factors that regulate IAV replication often show strain- or subtype-specific effects, reflecting intricate virus-host co-evolution (11, 12). Among these are DEAD-box RNA helicases (DDXs), which participate in diverse cellular RNA processes and antiviral defense (13, 14). Some DDXs, such as DDX58 (RIG-I), function as antiviral sensors (15). Others, including DDX3 and DDX6, are known to be exploited by various RNA viruses to promote replication (15, 16). Although several studies reported an interplay between IAV and DDXs (17–19), however, a systematic evaluation of whether different DDX proteins differentially regulate the replication of IAVs from distinct evolutionary backgrounds—particularly those with similar entry characteristics but divergent replication phenotypes—remains limited.

This knowledge gap is illustrated by pandemic H1N1 (H1N1pdm09) viruses and many contemporary avian-origin H9N2 viruses. Notably, some H9N2 lineages have evolved a human-type receptor-binding preference similar to H1N1pdm09 viruses (20, 21); however, they often show attenuated replication in human respiratory cells (22). This suggests that post-entry barriers, potentially involving polymerase function and vRNP assembly mediated by specific host factors, may limit H9N2 fitness in humans. Identifying host regulators that differentiate between such viruses could reveal key mechanisms underlying influenza polymerase adaptation.

Here, we performed a comparative screen of human DDX proteins to identify differential regulators of H1N1pdm09 and H9N2 polymerase activity. We found that DDX10 specifically regulates the replication and polymerase function of H1N1pdm09 viruses, but not a representative avian H9N2 strain. This functional specificity is mediated by residues 336 and 364 within the PB1 catalytic domain, which may be essential for a productive interaction with DDX10. Our work suggests a host-virus interface that differentially regulates influenza polymerase activity, providing molecular insight into how host factor compatibility influences the replication efficiency of distinct IAV subtypes in human cells.

## RESULTS

### DDX10 selectively regulates the polymerase activity and replication of influenza A H1N1 but not H9N2 viruses

**Fig 1:**
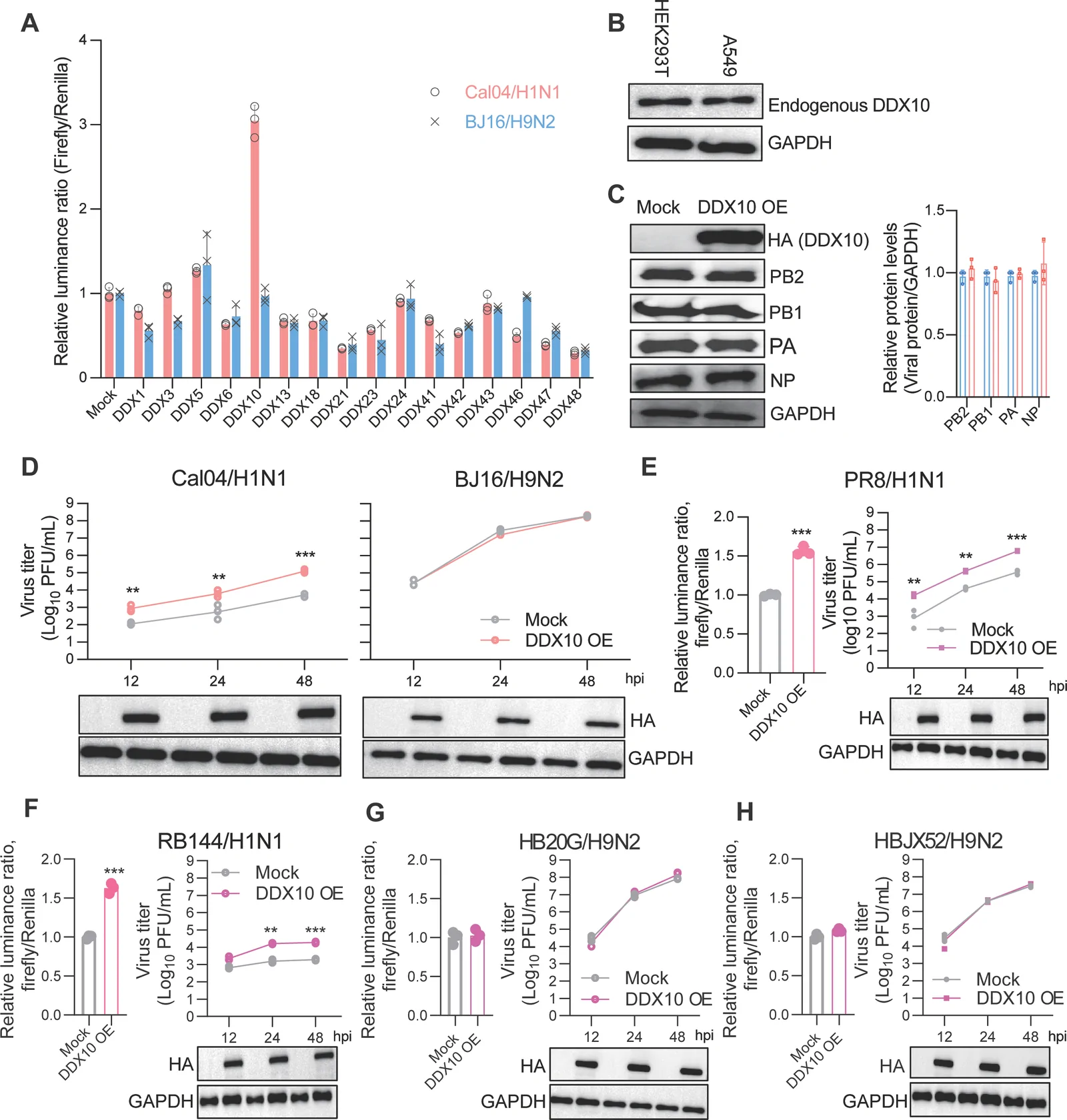
DDX10 selectively enhances the replication of H1N1, but not H9N2 influenza A viruses. (**A**) Screening of human DDX proteins for effects on viral polymerase activity. HEK293T cells were co-transfected with plasmids encoding the polymerase/NP components of Cal04/H1N1 or BJ16/H9N2, a pPolI-firefly luciferase reporter, a Renilla control, and individual Flag-tagged DDX plasmids (or empty pCAGGS). Normalized firefly luciferase activity was measured at 24 h post-transfection (hpt). (**B**) Endogenous DDX10 expression in HEK293T and A549 cells, analyzed by western blot (GAPDH as loading control). (**C**) Viral protein expression in the mini-genome system. HEK293T cells transfected with Cal04/H1N1 components plus HA-DDX10 or empty vector were immunoblotted for HA (DDX10), PB2, PB1, PA, and NP (left: representative blot; right: quantification normalized to GAPDH). (**D**) Virus growth kinetics. A549 cells transfected with HA-DDX10 or empty pCAGGS were infected with Cal04/H1N1 (left) or BJ16/H9N2 (right) (MOI = 0.005). Supernatants were titrated by plaque assay on MDCK cells at the indicated times. (**E and F**) Effect of DDX10 on other H1N1 strains. Left: polymerase activity of PR8/H1N1 and RB144/H1N1 in HEK293T cells transfected with HA-DDX10 or vector (pCAGGS) (luciferase assay, 24 hpt). Right: virus growth of PR8/H1N1 and RB144/H1N1 in A549 cells under the same conditions as in panel D. (**G and H**) Effect of DDX10 on other H9N2 strains. Left: polymerase activity of HB20G/H9N2 and HBJX52/H9N2 in HEK293T cells transfected with HA-DDX10 or empty pCAGGS (luciferase assay, 24 hpt). Virus growth of HB20G/H9N2 and HBJX52/H9N2 (right) in A549 cells, performed as in panel D. Overexpression of HA-DDX10 (**D–H**) was evaluated via western blotting with an HA tag-specific antibody. All graphs show mean ± s.d. of three independent experiments. Statistical significance was determined by two-way ANOVA or unpaired *t*-test, and only *P*-values less than 0.05 are marked, with significance levels indicated as **P < 0.01 and ***P < 0.001.

A phylogenetic analysis of the human DDX family members shows their evolutionary relationships (Fig. S1). We screened 16 representative members (DDX1, -3, -5, -6, -10, -13, -18, -21, -23, -24, -41, -42, -43, -46, -47, and -48) using a mini-genome (MG) reporter assay to determine if they differentially modulate the replication of human- and avian-origin IAVs. For this purpose, we employed two representative viral strains that share a preference for binding to human-type receptors but originate from different hosts: the pandemic A/California/04/2009 (H1N1) (Cal04/H1N1) and the zoonotic A/chicken/Beijing/16/2013 (H9N2) (BJ16/H9N2). This screen revealed that overexpression of DDX10 significantly regulated the activity of the human-origin viral polymerase but had no effect on the avian-origin viral polymerase (Fig. 1A), suggesting its differential role in modulating IAV replication from distinct host origins.

We confirmed comparable levels of endogenous DDX10 in HEK293T and A549 cells (Fig. 1B). Furthermore, overexpression of DDX10 did not affect the expression of viral polymerase subunits (PB2, PB1, and PA) or NP in the MG system (Fig. 1C), indicating that its modulatory effect may not be attributable to changes in viral protein expression.

Consistent with the MG assay results, overexpression of DDX10 in A549 cells significantly increased the titer of the Cal04/H1N1 virus but did not affect that of the BJ16/H9N2 virus (Fig. 1D). This strain-specific enhancement was also observed for other mammalian H1N1 strains: the polymerase activity (in 293T cells) and virus titers (in A549 cells) of both (A/Puerto Rico/8/1934 (H1N1) (PR8/H1N1) and A/swine/Hubei/RB144/2024 (H1N1) (RB144/H1N1)) were significantly upregulated upon DDX10 overexpression (Fig. 1E and F). In contrast, DDX10 overexpression did not enhance the polymerase activity in HEK293T cells and virus growth of other avian H9N2 strains, A/chicken/Hubei/20G/2025 (H9N2) (HB20G/H9N2) and A/chicken/Hubei/JX52/2025 (H9N2) (HBJX52/H9N2) (Fig. 1G and H).

### DDX10 knockdown impairs the replication of H1N1 but not H9N2 viruses

**Fig 2:**
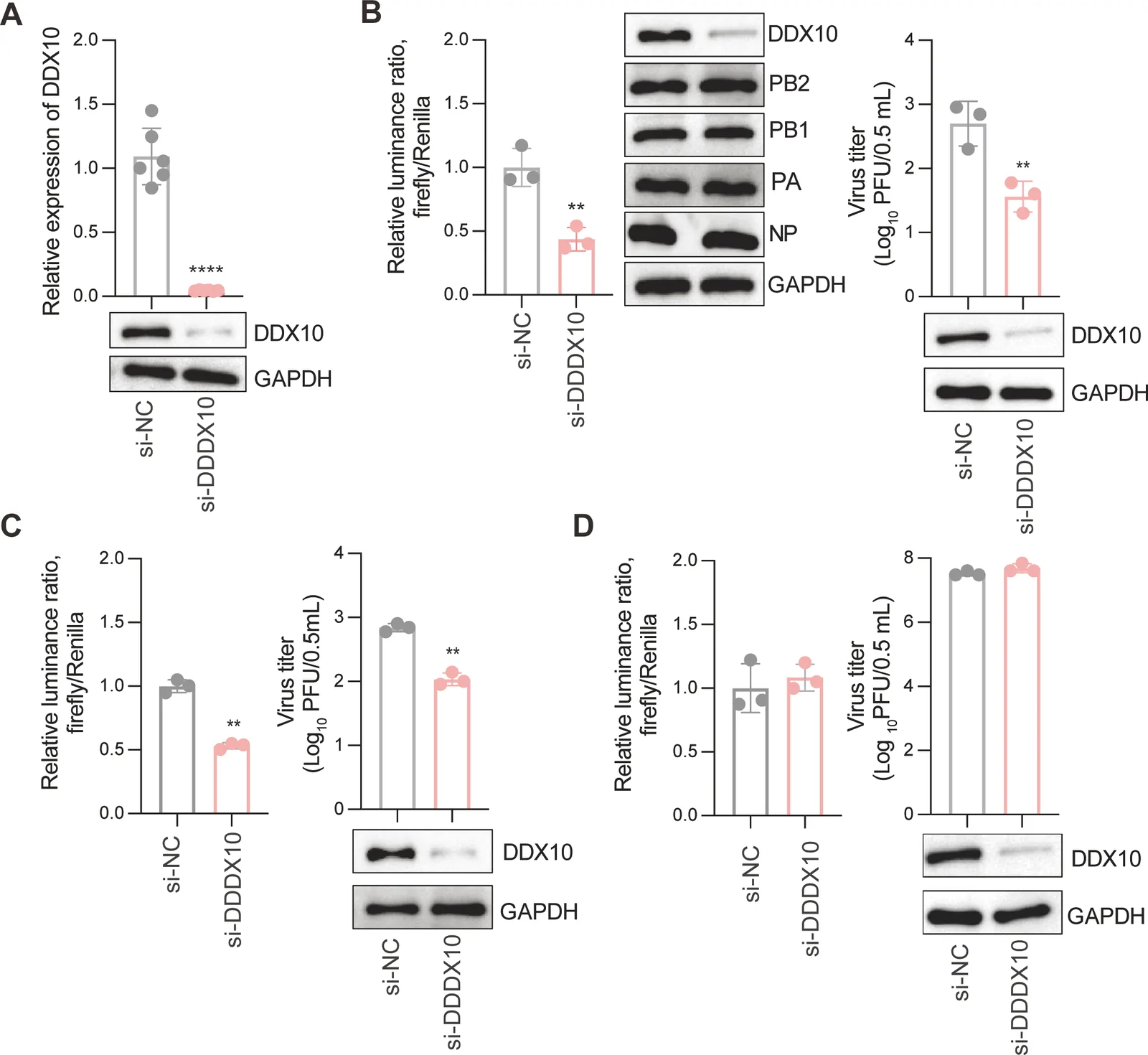
Knockdown of DDX10 impairs H1N1 but not H9N2 replication. (**A**) DDX10 knockdown validation in HEK293T cells transfected with si-DDX10 or a non-targeting control (si-NC), assessed by qRT-PCR (top) and western blot (bottom) at 24 hpt (GAPDH as loading control). (**B–D**) Effects of DDX10 knockdown on viral polymerase activity and replication. For each virus, HEK293T cells were co-transfected with si-DDX10 or si-NC and the corresponding mini-genome system; normalized luciferase activity was measured at 36 hpt (left panels). Viral protein (PB1, NP) and DDX10 levels were analyzed by western blot (middle panels). A549 cells transfected with si-DDX10 or si-NC were infected with the respective virus (MOI = 0.005); supernatants were collected at 24 hpi, and the virus titers were determined by plaque assay on MDCK cells (right panels, top). Knockdown efficiency in infected A549 cells was confirmed by western blot (right panels, bottom). Viruses tested: (**B**) Cal04/H1N1, (**C**) RB144/H1N1, and (**D**) BJ16/H9N2. All bar graphs show mean ± s.d. of three independent experiments. Statistical significance was determined by unpaired two-tailed Student’s *t*-test, and only *P*-values less than 0.05 are marked, with significance levels indicated as **P < 0.01 and ****P < 0.0001.

To further examine the role of endogenous DDX10, we knocked down the expression of endogenous DDX10 in HEK293T using siRNA (Fig. 2A), which did not affect cell viability ( Fig. S2). In these knockdown cells, both the polymerase activity and virus titers of the pandemic H1N1 virus (Cal04/H1N1) were significantly decreased (Fig. 2B). Similarly, the polymerase activity and virus titer of the swine-origin RB144/H1N1 isolate were also reduced (Fig. 2C). Conversely, knockdown of DDX10 did not significantly affect either the polymerase activity or the virus titer of the avian BJ16 H9N2 isolate (Fig. 2D). Consistent with this, DDX10 did not affect the production of viral polymerase subunits, a result observed for both the Cal04/H1N1 and BJ16/H9N2 viruses (Fig. 2B; Fig. S3). Collectively, these data demonstrate that DDX10 promotes IAV replication in a strain-specific manner, preferentially enhancing H1N1 viruses.

### The proviral activity of DDX10 is associated with its preferential binding to human-origin H1N1 PB1

**Fig 3:**
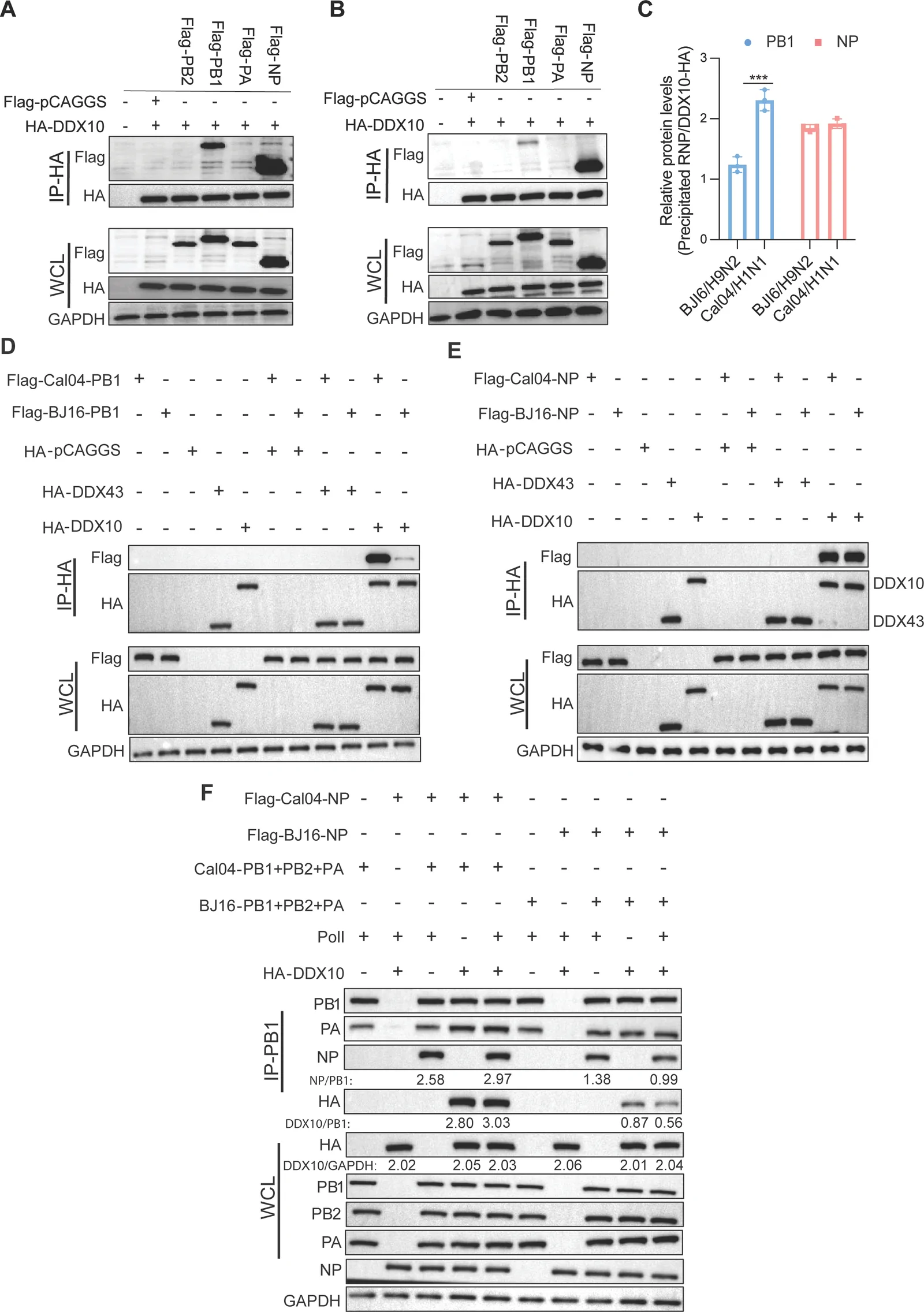
The proviral activity of DDX10 is associated with its preferential binding to human-origin (H1N1) PB1. (**A and B**) Co-immunoprecipitation (Co-IP) of DDX10 with vRNP subunits. HEK293T cells were co-transfected with HA-DDX10 and Flag-tagged PB2, PB1, PA, or NP from Cal04/H1N1 (**A**) or BJ16/H9N2 (**B**). Cell lysates were subjected to anti-HA IP, followed by western blotting with the indicated antibodies. WCL, whole-cell lysate. (**C**) Quantification of interaction strength. Band intensities of co-precipitated PB1 and NP from panels A and B were measured (ImageJ). Bars show mean ± s.d. (*n* = 3). *P*-value less than 0.05 is marked, with significance levels indicated as ***P < 0.001 (unpaired *t*-test). (**D**) Specificity of the PB1-DDX10 interaction. HEK293T cells were co-transfected with Flag-PB1 (Cal04/H1N1 or BJ16/H9N2) and either empty HA-pCAGGS, HA-DDX43, or HA-DDX10. HA pull-down was then performed, followed by western blotting with the indicated antibodies. (**E**) Specificity of the NP-DDX10 binding. HEK293T cells were co-expressed with Flag-NP (Cal04/H1N1 or BJ16/H9N2) and either empty HA-pCAGGS, HA-DDX43, or HA-DDX10. HA pull-down was then performed, followed by western blotting with the indicated Flag and HA tag-specific antibodies. (**F**) HEK293T cells were co-transfected with RNP plasmids (Cal04/H1N1 or BJ16/H9N2) and HA-DDX10. Cell lysates were immunoprecipitated with anti-PB1 antibody and blotted with the indicated antibodies. Band intensities of co-precipitated HA-DDX10 and NP and input HA-DDX10 were measured (ImageJ). All blots are representative of three independent experiments.

To elucidate how DDX10 regulates IAV replication in a strain-specific manner, we attempted to examine the potential interactions between DDX10 and the viral ribonucleoprotein (vRNP) components. Co-immunoprecipitation (Co-IP) assays in HEK293T cells, co-transfected with HA-DDX10 and Flag-tagged PB2, PB1, PA, or NP (from either Cal04/H1N1 or BJ16/H9N2) showed that DDX10 specifically co-precipitated with PB1 and NP, but not with PB2 or PA, from both viral strains (Fig. 3A and B). Notably, the interaction with Cal04/H1N1 PB1 was markedly stronger than with BJ16/H9N2 PB1, whereas the binding of DDX10 to the NP of the H1N1 and H9N2 viruses is more indicative of non-specific binding (Fig. 3C). Further Co-IP assays, including appropriate controls with empty HA-pCAGGS and an unrelated protein (DDX43 and DDX24, which did not affect viral polymerase activity), confirmed that PB1 and NP specifically interact with DDX10, ruling out background binding (Fig. 3D and E; Fig. S4A). These results indicate that the strain-specific effect of DDX10 is determined by its differential interaction with PB1, rather than NP.

To determine whether RNA mediates these interactions, lysates from cells co-expressing HA-DDX10 with Flag-PB1 or Flag-NP (Cal04/H1N1), both with and without the complete vRNP complex, were treated with RNase-A prior to Co-IP. DDX10 still co-precipitated with PB1 and NP after RNase treatment (Fig. S4B), confirming RNA-independent binding. We next asked whether DDX10 associates with fully assembled vRNP complexes. Based on a previously described strategy (23), a vRNP reconstitution system in HEK293T cells was co-transfected with PB2, PB1, PA, NP, a pPol I plasmid encoding an untranslated viral minigenome, and HA-tagged DDX10. Successful vRNP assembly was confirmed by the co-precipitation of NP with PB1. DDX10 was also present in the immunoprecipitated complex, demonstrating its association with the assembled vRNP. Importantly, DDX10 showed stronger binding affinity with Cal04/H1N1 RNP than with BJ16/H9N2 RNP (Fig. 3F).

### DDX10 facilitates viral polymerase complex formation and RNP assembly in a strain-specific manner

**Fig 4:**
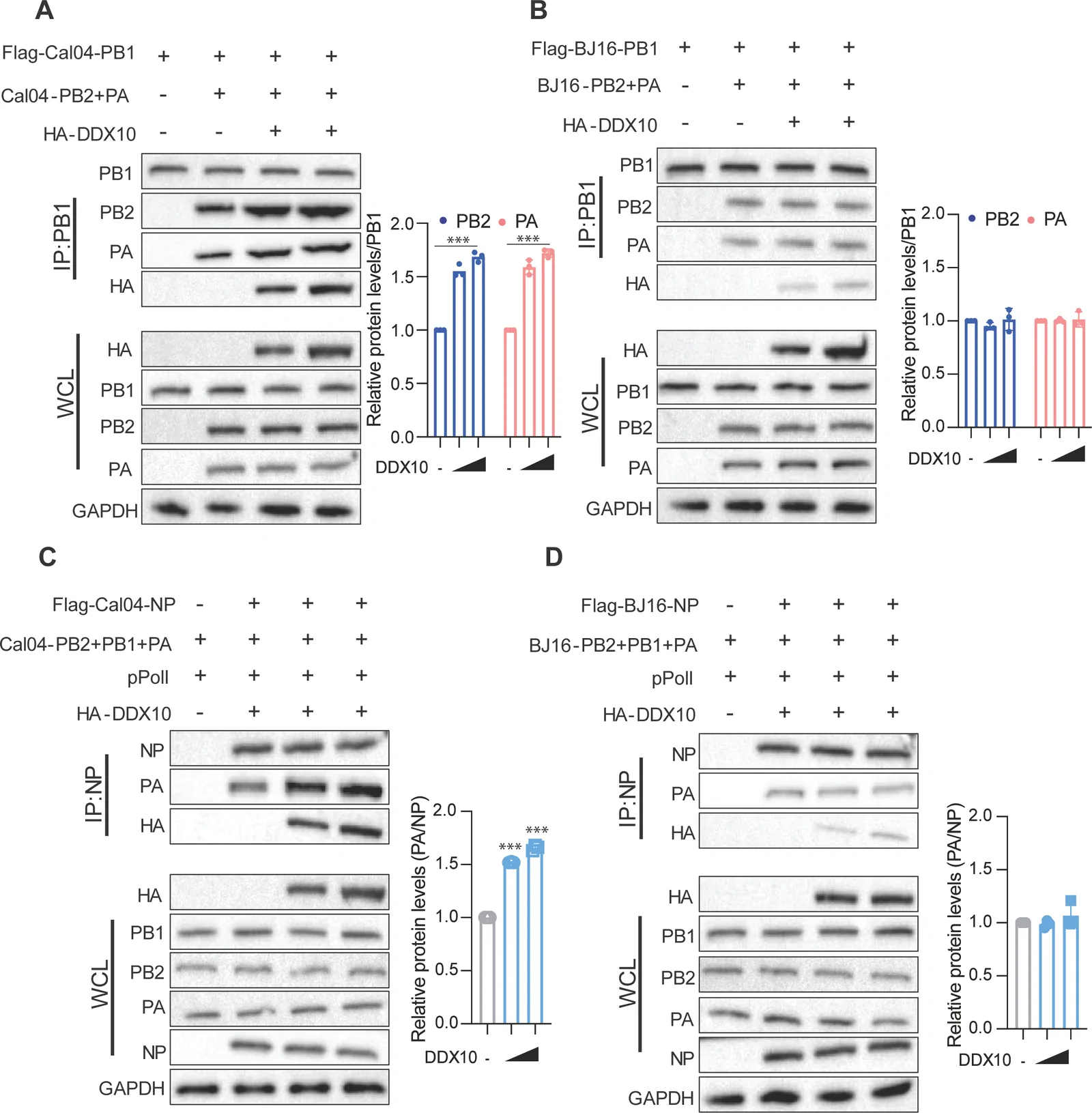
DDX10 facilitates viral polymerase trimer formation and vRNP assembly in a strain-specific manner. (**A and B**) DDX10 enhances polymerase complex assembly. HEK293T cells were co-transfected with plasmids expressing Flag-tagged PB2, PB1, and PA from Cal04/H1N1 (**A**) or BJ16/H9N2 (**B**), together with increasing amounts (0, 1.0, or 1.5 µg) of HA-DDX10 plasmid. At 36 hpt, cell lysates were subjected to immunoprecipitation (IP) using an anti-PB1 antibody. Co-precipitated PB2 and PA were detected by western blotting (left panels). Band intensities were quantified, and the ratios of co-IP PB2/PB1 and PA/PB1 are plotted (right panels). (**C and D**) DDX10 promotes complete vRNP assembly. HEK293T cells were co-transfected with plasmids encoding full vRNP components (Flag-PB2, -PB1, -PA, and -NP) from Cal04/H1N1 (**C**) or BJ16/H9N2 (**D**), together with increasing HA-DDX10 (0, 1.0, and 1.5 µg). IP was performed using an anti-NP antibody. Co-precipitated PA (vRNP assembly marker) and DDX10 were detected by blotting (left panels). The PA/NP intensity ratios are shown (right panels). All graphs show mean ± s.d. from three independent experiments. Statistical significance was determined by one-way ANOVA with Dunnett’s test (compared to the 0 µg DDX10 group**;** *P*-values less than 0.05 are marked, with significance levels indicated as ***P < 0.001)*.*

To determine how the DDX10-PB1 interaction affects the viral life cycle, we assessed its role in viral ribonucleoprotein (vRNP) assembly, a critical step for IAV’s replication (24). While DDX10 overexpression did not affect NP oligomerization (Fig. S5), Co-IP assays showed that DDX10 strengthened the interaction between PB1 and its partner subunits PB2 and PA in the Cal04/H1N1 polymerase complex (Fig. 4A), but not in the BJ16/H9N2 polymerase (Fig. 4B). Furthermore, in a vRNP reconstitution assay, DDX10 dose-dependently increased the amount of PA co-precipitated with NP, a key indicator of complete vRNP assembly for Cal04/H1N1 (Fig. 4C), but not for BJ16/H9N2 (Fig. 4D). These results indicate that DDX10 facilitates the formation of the polymerase trimer and its subsequent integration with NP-coated vRNA into functional vRNPs, specifically in viruses that are responsive to DDX10.

### Residues 336 and 364 within the PB1 catalytic domain are critical for DDX10-mediated enhancement

**Fig 5:**
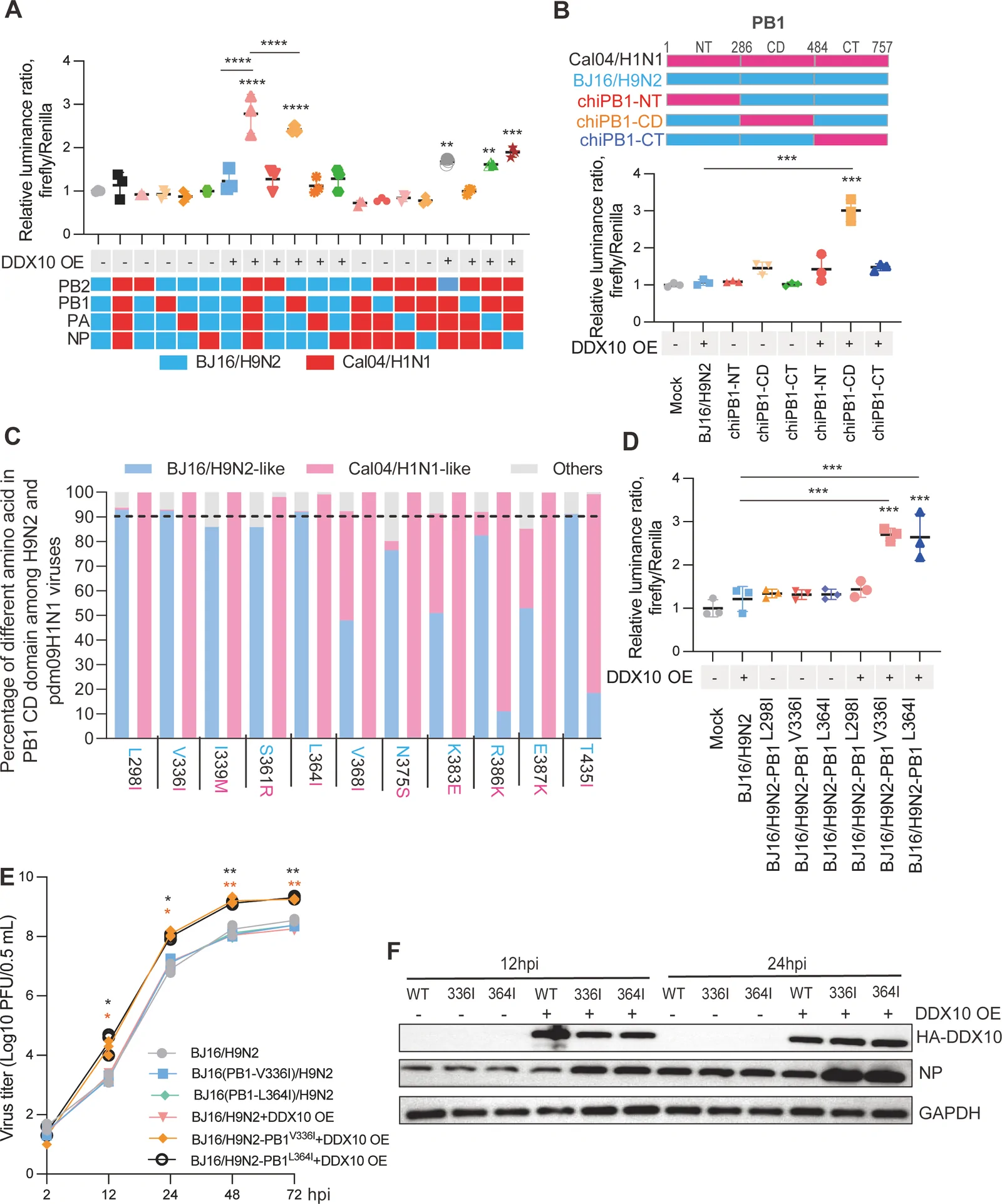
Residues 336 and 364 in the PB1 catalytic domain govern strain-specific enhancement by DDX10. (**A**) Identification of PB1 as the key determinant. Polymerase activity of BJ16/H9N2 vRNPs containing individual segments swapped with Cal04/H1N1 was measured in HEK293T cells upon DDX10 overexpression. Activity is shown relative to the parental BJ16/H9N2 vRNP (set to 1). (**B**) Mapping the critical region. (Top) Schematic of chimeric PB1 proteins generated by swapping the N-terminal (NT), catalytic domain (CD), or C-terminal (CT) between Cal04/H1N1 and BJ16/H9N2 PB1. (Bottom) Polymerase activity of BJ16/H9N2 vRNPs bearing each chimeric PB1, with or without DDX10 overexpression. (**C**) Residue frequency analysis within the PB1-CD. Amino acid frequencies at divergent positions were analyzed between H1N1pdm09 and H9N2 sequences. A dashed line at 90% specifies the cutoff threshold for selecting the three mutants. (**D**) Validation of key residues. (Top) Alignment of the PB1-CD from Cal04/H1N1 and BJ16/H9N2, highlighting 11 divergent residues; three positions (298, 336, and 364) with high strain-specificity were selected. (Bottom) Polymerase activity of BJ16/H9N2 vRNPs containing the indicated PB1 single-point mutations (L298I, V336I, and L364I) with DDX10 overexpression. (**E**) Growth enhancement by PB1 mutations. Growth kinetics of wild-type BJ16/H9N2 and isogenic mutants (PB1-V336I and PB1-L364I) in A549 cells with or without DDX10 overexpression (MOI = 0.005). Virus titers were determined by plaque assay. (**F**) Viral protein accumulation. NP levels in A549 cells infected as in panel E were analyzed by western blot at 12 and 24 hpi (GAPDH as loading control). Data are mean ± s.d. of three independent experiments. Statistical significance was determined by two-way ANOVA or unpaired *t*-test. *P*-values less than 0.05 are marked, with significance levels indicated as *P < 0.05, **P < 0.01, ***P < 0.001, and ****P < 0.0001.

To identify the viral determinant of DDX10’s strain specificity, we constructed a panel of reassortment vRNPs by systematically swapping individual polymerase/NP proteins between the BJ16/H9N2 and Cal04/H1N1 backbones. When DDX10 was overexpressed, only the reassorted vRNP containing Cal04/H1N1 PB1 within the BJ16/H9N2 background exhibited significantly enhanced polymerase activity; replacement of other segments (PB2, PA, or NP) did not restore this enhancement (Fig. 5A). Conversely, introducing BJ16/H9N2-PB1 into the Cal04/H1N1 background abrogated the DDX10-mediated increase in polymerase activity (Fig. 5A). These results demonstrate that PB1 is the critical viral subunit responsible for the differential regulation by DDX10.

We next aligned the PB1 sequences of Cal04/H1N1 and BJ16/H9N2, identifying 34 amino acid differences between them (Table S1). To map the region responsible for the differential DDX10 interaction, we generated chimeric PB1 proteins and tested them in the mini-genome assay. This approach localized the functional determinant to the central catalytic domain (CD) of PB1 (Fig. 5B). Within the CD, which contains 11 divergent residues, bioinformatic analysis highlighted three positions (298, 336, and 364) exhibiting high host-specific residue frequencies (>90%) between H1N1pdm09 and avian H9N2 sequences (Fig. 5C). Structural analysis indicated that all three residues are located on the surface of the PB1 protein (Fig. S6).

**Fig 6:**
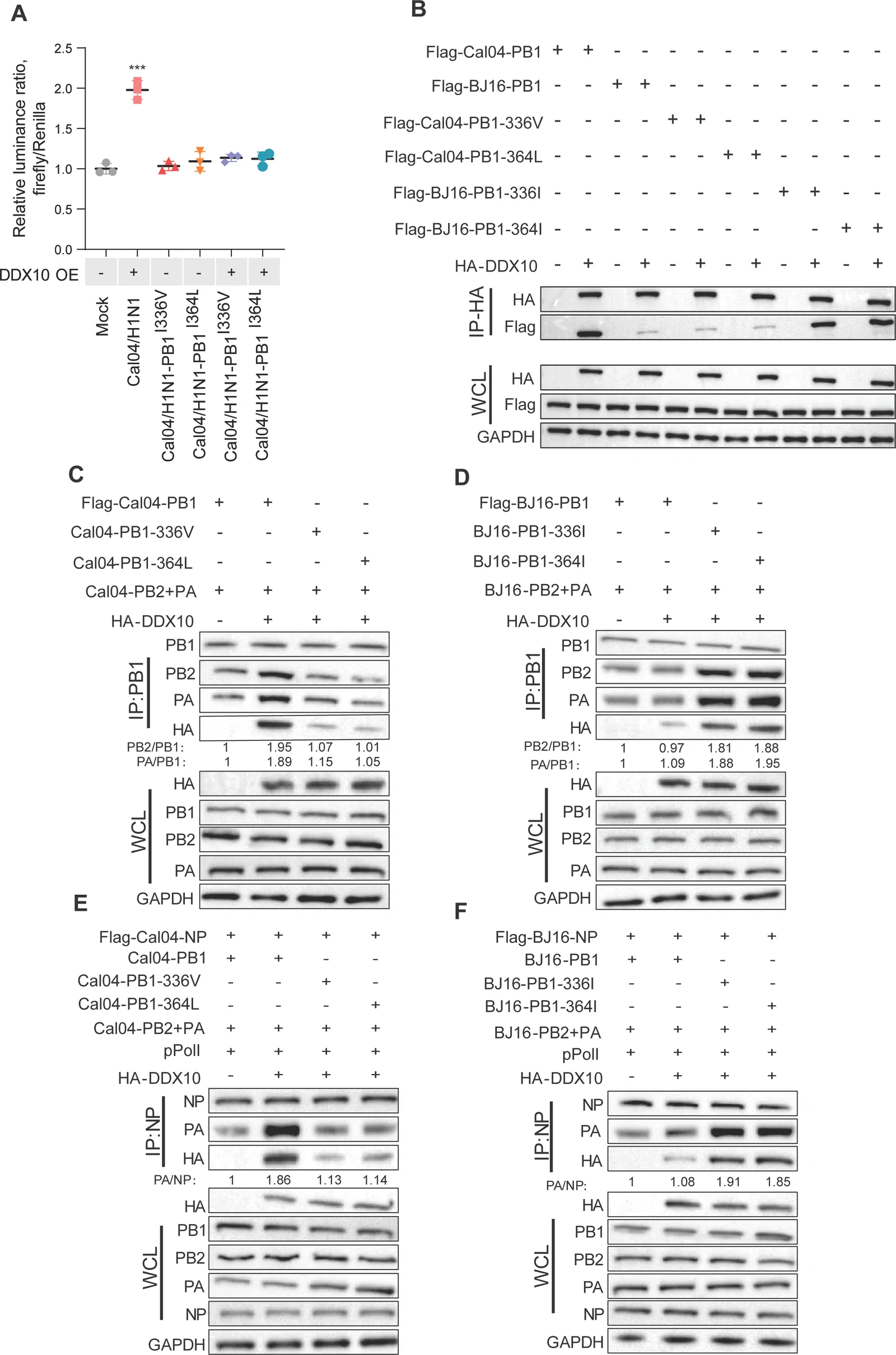
Reciprocal validation of PB1 residues 336 and 364 in conferring DDX10 responsiveness. (**A**) Loss-of-function mutations in Cal04/H1N1 PB1. HEK293T cells were co-transfected with DDX10 and the Cal04/H1N1 mini-genome system containing wild-type PB1 or PB1 with the indicated reverse mutations (I336V, I364L). Normalized polymerase activity is shown. (**B**) Impact of PB1 mutations on interaction with DDX10. Co-IP assay was performed in HEK293T cells co-expressing HA-DDX10 and Flag-tagged wild-type or mutant PB1 (I336V, I364L, V336I, and L364I), using an anti-HA antibody. Co-precipitated PB1 was detected by western blot. (**C and D**) DDX10 promotes polymerase complex formation in a strain-dependent way. HEK293T cells were transfected with Flag-PB2, Flag-PB1 (wild-type or 336/364 mutants from Cal04/H1N1 [**C**] or BJ16/H9N2 [**D**]), and PA, along with HA-DDX10. IP with anti-PB1 shows co-precipitated PB2 and PA. Band intensities were quantified, and the ratios of co-IP PB2/PB1 and PA/PB1 are shown. (**E and F**) DDX10 facilitates vRNP assembly depending on PB1 residues 336 and 364. HEK293T cells were transfected with full vRNP components (PB2, PB1, PA, and NP-Flag) from Cal04 with PB1-336V/PB1-364L (**E**) or BJ16 (**F**) with PB1-336I/364I, plus HA-DDX10. IP with anti-NP detects co-precipitated PA (PA/NP ratio shown). Data are mean ± s.d. of three independent experiments. Statistical significance was determined by two-way ANOVA. *P*-value less than 0.05 is marked, with significance levels indicated as ***P < 0.001*.*

We then introduced single H1N1-type substitutions (L298I, V336I, and L364I) into BJ16/H9N2 PB1. The V336I and L364I mutations, but not L298I, significantly enhanced polymerase activity upon DDX10 overexpression (Fig. 5D). Similarly, in the context of recombinant virus, both V336I and L364I significantly increased BJ16/H9N2 virus titers and viral protein (NP) levels only when DDX10 was overexpressed (Fig. 5E and F). In contrast, the reverse mutations (I336V and I364L) in Cal04/H1N1 PB1 significantly decreased the DDX10-mediated increase in polymerase activity and weakened the interaction with DDX10 (Fig. 6A and B).

Moreover, DDX10 promoted viral polymerase complex formation and vRNP assembly depending on PB1 residues 336 and 364. In Cal04/H1N1, DDX10 enhanced polymerase complex formation and vRNP assembly only when wild-type PB1 was present; mutations at residues 336V and 364L abolished this effect. Conversely, in BJ16/H9N2, DDX10 enhanced polymerase complex and vRNP assembly specifically in the presence of the PB1 336I and 364I mutants but not with wild-type PB1 (Fig. 6C through F). These data indicate that residues 336 and 364 within the PB1 catalytic domain are critical for DDX10-mediated enhancement of virus replication.

### Natural variation of PB1 residues 336 and 364 across influenza A viruses

To place our mechanistic findings in an epidemiological context, we analyzed the natural variation of PB1 residues 336 and 364 using publicly available sequences of influenza A viruses from human, avian, and swine hosts, spanning subtypes H1N1, H3N2, H5N1, H7N9, and H9N2.

**Fig 7:**
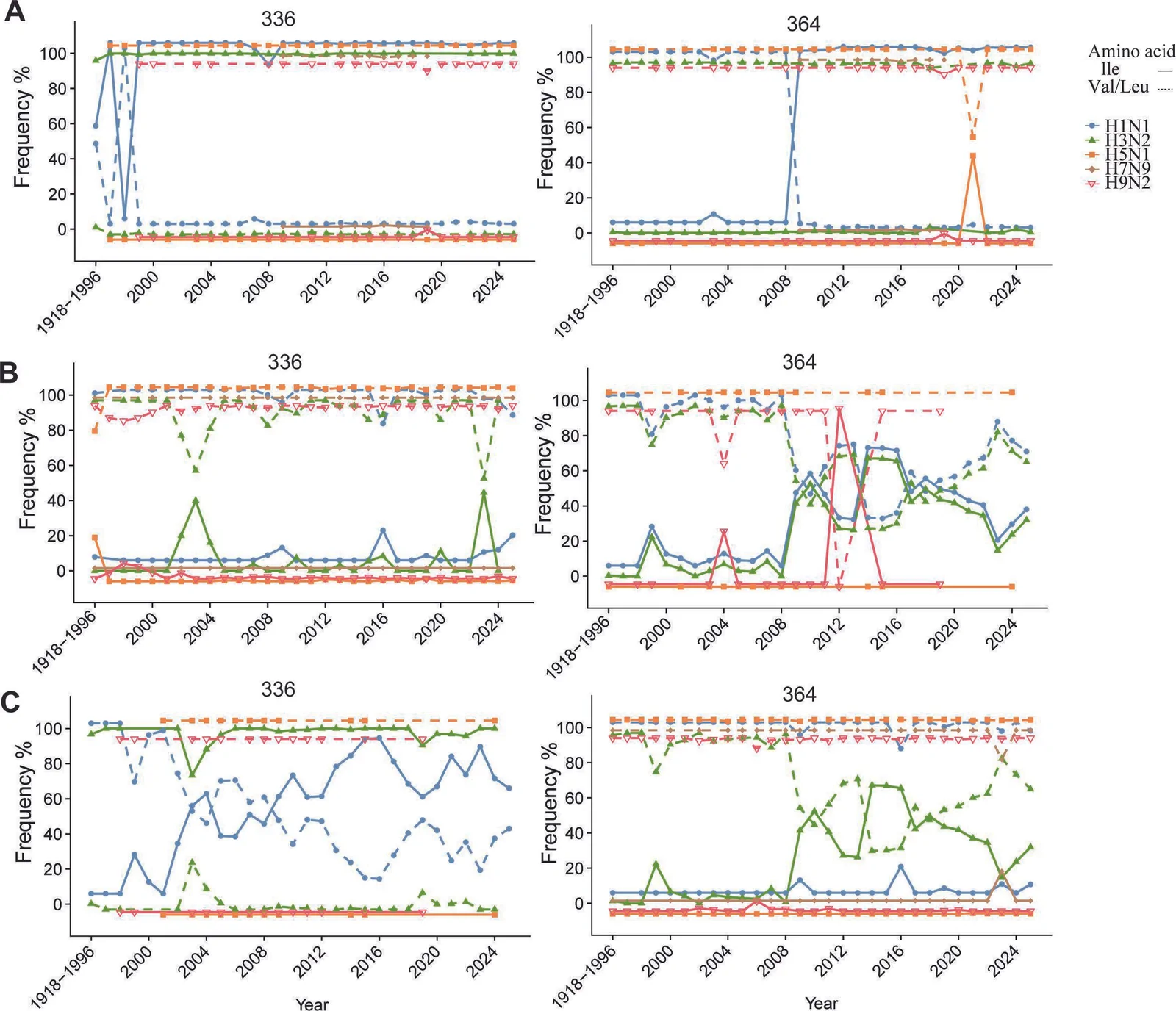
Host-specific patterns of PB1 residues 336 and 364 in influenza A viruses. (**A–C**) Frequencies of amino acids at positions 336 and 364 in PB1 protein sequences from (**A**) human-, (**B**) avian-, and (**C**) swine-origin influenza A viruses (subtypes H1N1, H3N2, H5N1, H7N9, and H9N2). Sequences were retrieved from public databases (up to 1 January 2026) and analyzed as described in Materials and Methods.

A clear host-associated pattern emerged. In human-adapted lineages, isoleucine (Ile) was overwhelmingly dominant at both positions 336 and 364 (Fig. 7A). For human H1N1 viruses, Ile was present at residues 336 and 364 in >99% and >91.7% of sequences, respectively. Similarly, in human H3N2 viruses, Ile was found in >99% of sequences at position 336, while >97.5% Leu at position 364.

In contrast, avian viruses across all analyzed subtypes (H1N1, H5N1, H7N9, and H9N2) predominantly carried valine (Val) at position 336 and leucine (Leu) at position 364, with each exceeding 90% frequency in most surveillance years (Fig. 7B). This Val336/Leu364 combination appears to be the major avian signature. Minor exceptions were noted; for instance, approximately 15%–17% of avian H1N1 sequences from 2016 contained Ile at one or both positions.

Viruses isolated from swine exhibited an intermediate or mixed profile (Fig. 7C). Swine H1N1 viruses showed co-circulation of Val and Ile at position 336 and of Leu and Ile at position 364. The swine H1N1 strain (A/swine/Hubei/RB144/2024) used in our study, which carries Ile at both residues, reflects this polymorphism. Swine H3N2 viruses also displayed variability at these sites across different years.

In summary, Ile336 and Ile364 constitute a signature highly conserved in human influenza virus lineages, whereas Val336 and Leu364 are predominant in avian viruses. The occurrence of human-type residues in a subset of avian and swine isolates indicates that these positions are naturally variable, and such substitutions may influence viral fitness in different hosts.

### DDX10 expression is higher in A549 cells than in primary airway epithelial cells, limiting the predicted risk of H9N2 PB1 mutants

The present study supports the finding that viral replication is favored only when DDX10 is overexpressed. To evaluate whether other human lung cells naturally express higher levels of DDX10, we assessed endogenous DDX10 expression in A549 cells compared to other primary human lung cells that are natural targets of influenza virus infection. Both qPCR and western blotting revealed that DDX10 expression in A549 cells is higher than that in Calu-3 (human lung epithelial) and NHBE (normal human bronchial epithelial) cells (Fig. S7A and B). These results argue against the possibility that DDX10 serves as a host-range determinant that can support the replication of viruses carrying human-like residues at positions 336 and 364 under physiological expression levels. However, further studies are needed to fully assess the potential risks associated with these mutations in physiologically relevant models.

## DISCUSSION

This study demonstrates that the host RNA helicase DDX10 differentially regulates influenza A virus replication by interacting with the viral polymerase in a strain-specific manner. We found that DDX10 selectively enhances the polymerase activity and replication of human-adapted H1N1 viruses, including the 2009 pandemic strain, but not an avian-origin H9N2 virus. This specificity is mediated by a direct interaction between DDX10 and the viral PB1 subunit, dependent on two residues (336 and 364) within the PB1 catalytic domain. These findings define a specific host-virus interface that contributes to the replication efficiency of IAVs in human cells.

DDX10 is a DEAD-box RNA helicase primarily involved in ribosome biogenesis (25). While other DDX proteins, such as DDX3, are known to support various viruses (26–28), a role for DDX10 in IAV replication had not been reported. Our results extend the understanding of how viruses utilize host RNA helicases and reveal functional selectivity within this protein family. The strain-specific activity of DDX10 is consistent with the behavior of other host factors, such as ANP32 proteins, which also show preferential support for human-adapted viral polymerases (29, 30). This highlights how discrete molecular compatibilities can influence viral fitness in a new host.

The viral polymerase subunit PB1 was identified as the critical determinant for DDX10 responsiveness. PB1 carries the core catalytic functions for replication and is a major factor in viral adaptation; its acquisition from avian viruses has been a feature of past pandemics (31). Unlike surface glycoproteins that mediate receptor binding and immune evasion, PB1 modulates the core replication machinery (31–34). Host factors can interact with PB1 to either inhibit (e.g., via TRIM32-mediated ubiquitination) (35) or promote (e.g., by protecting it from degradation) (36) viral replication. Our study places DDX10 in the latter category and identifies a specific mechanism—facilitating polymerase and vRNP assembly—by which it enhances PB1 function.

It is worth noting that the DDX10 interaction with PB1 is not absolutely species-restricted, but DDX10 shows stronger binding affinity toward human-origin PB1 (Cal04/H1N1) compared to avian-origin PB1 (BJ16/H9N2). The molecular basis for this differential interaction was mapped to residues 336 and 364 in PB1, which correlate with the strain-specific enhancement of polymerase activity. The H1N1-type residues (I336 and I364) enable a productive interaction with DDX10, leading to enhanced replication. In contrast, the common avian-type residues (V336 and L364) in H9N2-PB1 result in a DDX10-insensitive phenotype. However, we cannot exclude the possibility that other residues or structural components of the polymerase complex also influence DDX10 association and consequently this effect. Analysis of public sequence data indicates that V336I and L364I substitutions have emerged in PB1 of some circulating H9N2 viruses. This suggests a potential adaptive path: as H9N2 viruses circulate, they may acquire these mutations, potentially gaining the ability to utilize the human host factor DDX10 to improve their replication in mammalian cells. Monitoring these PB1 residues could therefore serve as a genetic marker for the adaptation potential of zoonotic H9N2 strains. Moreover, the current study reported higher DDX10 levels in A549 cells than primary human airway epithelial cells (Calu-3 and NHBE); the elevated DDX10 expression in this cell line may favor the proviral effects observed here and may not fully reflect the physiological context of the human respiratory tract. Our data suggest that the enhanced viral growth, including that of the H9N2 mutants (V336I and L364I), may be conditional upon high DDX10 expression levels. Since primary airway cells have lower endogenous DDX10 levels, it remains unknown to what extent these effects occur during natural infection. Therefore, additional research using primary human airway epithelial cells, *ex vivo* lung models, or suitable animal models is necessary to determine the true physiological relevance of our findings and the potential risks associated with these mutations. Until such studies are conducted, the implications of our findings for natural infection and transmission remain uncertain.

From a translational perspective, the DDX10-PB1 interaction interface represents a potential target for antiviral development. Its strain-specific nature raises the possibility of designing agents that selectively disrupt the replication of DDX10-dependent viruses. Future work should aim to determine the structural details of the DDX10-PB1 complex and investigate whether modulating this interaction influences viral polymerase function and evolution.

In summary, we describe a mechanism in which the host RNA helicase DDX10 is co-opted by influenza viruses in a strain-specific manner to promote replication. This specificity is governed by a two-residue signature in the PB1 catalytic domain, revealing a defined host-pathogen interaction that affects viral fitness. These findings contribute to understanding how influenza viruses adapt to human hosts and provide specific genetic markers for surveilling avian viruses with zoonotic potential.

## MATERIALS AND METHODS

### Cells

Human embryonic kidney 293T cells (HEK293T), Madin-Darby canine kidney (MDCK) cells, and human alveolar adenocarcinoma (A549) cells were purchased from the American Type Culture Collection (ATCC). HEK293T cells were cultured in Dulbecco’s modified Eagle medium (DMEM; Gibco) supplemented with 10% fetal bovine serum (FBS; Gibco) and 1% penicillin/streptomycin (P/S; Gibco). A549 cells were cultured in DMEM/F-12 (Gibco) supplemented with 10% fetal bovine serum (FBS; Gibco) and 1% penicillin/streptomycin (P/S; Gibco). MDCK cells were maintained in minimum essential medium (MEM; Gibco) containing 10% FBS, 1% P/S, 1% non-essential amino acids (Gibco), and 25 mM HEPES (Gibco). All cells were incubated at 37°C in a humidified 5% CO2 atmosphere.

### Viruses

Influenza A viruses used in this study were as follows: A/Puerto Rico/8/1934 (H1N1) (PR8/H1N1), A/California/04/2009 (H1N1) (Cal04/H1N1), A**/**swine/Hubei/RB144/2024 (H1N1) (RB144/H1N1), A**/**chicken/Beijing/16/2013 (H9N2) (BJ16/H9N2), A/chicken/Hubei/JX52/2025 (H9N2) (HBJX52/H9N2), and A/chicken/Hubei/20G/2025 (H9N2) (HB20G/H9N2). Mutants (BJ16-PB1-V336I and BJ16-PB1-L364I) were rescued using plasmid-based reverse genetics and verified by Sanger sequencing. Viruses used in this study are classified as low-pathogenicity strains, as confirmed by the presence of a monobasic cleavage site in the hemagglutinin protein (37).

### Plasmid construction

The open reading frames of human DDX10 and influenza viral proteins (PB2, PB1, PA, and NP) were amplified by PCR and cloned into the mammalian expression vector pCAGGS. An N-terminal HA or Flag tag was added to each construct using standard cloning methods. Chimeric PB1 plasmids (chiPB1-NT, -CD, and -CT) were generated by swapping the corresponding domains between the Cal04/H1N1 and BJ16/H9N2 PB1 sequences (Table S2). Single-amino-acid substitutions (L298I, V336I, and L364I in BJ16/H9N2-PB1; I336V and I364L in Cal04/H1N1-PB1) were introduced by overlap-extension PCR. All constructs were verified by Sanger sequencing.

### Antibodies and reagents

The following primary antibodies were used: rabbit anti-DDX10 (Proteintech, 17857-1-AP), mouse anti-HA (MBL, M180-3), mouse anti-Flag (MBL, M185-3L), rabbit anti-PB2 (GeneTex, GTX125926), rabbit anti-PB1 (GeneTex, GTX125923), rabbit anti-PA (GeneTex, GTX118991), mouse anti-NP (GeneTex, GTX629633), and mouse anti-GAPDH (Proteintech, 60004-1-Ig). For western blotting, horseradish peroxidase (HRP)-conjugated goat anti-rabbit IgG (Beyotime, A0208) and goat anti-mouse IgG (Beyotime, A0216) served as secondary antibodies. For immunofluorescence, Alexa Fluor 488-conjugated goat anti-mouse IgG (Abbkine, A23210) and Alexa Fluor 594-conjugated goat anti-rabbit IgG (Abbkine, A23420) were used.

### Mini-genome reporter assay

A luciferase-based mini-genome system was used to assess viral polymerase activity. HEK293T cells seeded in 24-well plates were co-transfected using jetPRIME (Polyplus) with the following plasmids: viral polymerase subunits and NP (PB2, 80 ng; PB1, 80 ng; PA, 40 ng; and NP, 160 ng), a pPolI-driven firefly luciferase reporter (80 ng), a Renilla luciferase control (pRL-TK, 20 ng), and either a DDX-expression plasmid or an empty vector (250 ng) in triplicate. At 24 h post-transfection, cells were lysed, and firefly luciferase activity was measured with a dual-luciferase assay kit (Promega) and normalized to the Renilla luciferase signal. The entire experiment was independently repeated two to three times.

### siRNA-mediated knockdown

For DDX10 knockdown, HEK293T cells were transfected with 20 nM of a previously validated siRNA targeting DDX10 (sense: 5′-AUUCUUUCCUACUUUUCGGAG-3′) or a non-targeting control siRNA (Sangon Biotech) using jetPRIME (25). Knockdown efficiency was assessed at 24 hpt by qRT-PCR and western blotting.

### qPCR

Total RNA was extracted from cells using TRIzol reagent (Invitrogen) according to the manufacturer’s protocol. cDNA was synthesized from 1 µg of RNA using random primers and reverse transcriptase (Promega) in a 20 µL reaction. Quantitative PCR was performed with the resulting cDNA using gene-specific primers and AceQ qPCR SYBR Green Master Mix (Vazyme) on a Roche LightCycler 96. The cycling conditions were as follows: 95°C for 5 min, followed by 40 cycles of 95°C for 10 s and 60°C for 30 s. Relative mRNA levels were calculated by the 2^−ΔΔCt^ method using GAPDH as the internal reference.

### Western blotting

Cells were lysed in RIPA buffer (Beyotime) supplemented with a protease inhibitor cocktail (Beyotime). Protein lysates were separated by SDS-PAGE and transferred to PVDF membranes. After blocking with 5% skim milk, membranes were incubated with primary antibodies overnight at 4°C, followed by HRP-conjugated secondary antibodies for 1 h at room temperature. Signals were detected using an enhanced chemiluminescence substrate (Bio-Rad).

### Co-immunoprecipitation (Co-IP)

HEK293T cells were transfected with relevant expression plasmids in triplicate. At 36 hpt, cells were lysed in IP buffer (Beyotime). For immunoprecipitation, clarified lysates were incubated overnight at 4°C with anti-HA or anti-Flag antibody, followed by incubation with Protein A/G beads (Beyotime) for 4 h. Beads were washed three times with IP buffer, and bound proteins were eluted with 1× SDS loading buffer for subsequent western blot analysis. The entire experiment was independently repeated three times.

### vRNP reconstitution assay

To examine the interaction of DDX10 with assembled viral ribonucleoprotein (vRNP) complexes, a reconstitution system was used. HEK293T cells were co-transfected with plasmids expressing influenza polymerase subunits (PB2, PB1, and PA), NP, an untranslated viral minigenome (driven by pPol I), and HA-tagged DDX10 in triplicate. At 36 h post-transfection, cell lysates were incubated with an anti-PB1 antibody. The precipitated complexes were then analyzed by western blotting to detect co-associated DDX10 and vRNP components.

### Virus growth kinetics

Virus replication was assessed by growth curve analysis. A549 cells were transfected with either HA-DDX10 or an empty vector control (pCAGGS) using Lipofectamine 3000 (Invitrogen) according to the manufacturer’s instructions. Briefly, Lipofectamine 3000 Reagent was diluted in Opti-MEM Medium (Gibco) and mixed with a master mix of plasmids prepared in Opti-MEM Medium containing P3000 Reagent. The mixture was incubated at room temperature, allowing DNA-lipid complex formation, and then added to the cells. At 24 h post-transfection, cells were infected with influenza virus at a multiplicity of infection (MOI) of 0.005, in triplicate. After a 1-h adsorption period, the inoculum was removed and replaced with infection medium (DMEM/F-12 containing 1 µg/mL TPCK-treated trypsin). Supernatants were harvested at the indicated time points, and virus titers were determined by plaque assay on MDCK cells. The entire experiment was independently repeated three times.

### Cell viability assay

Cell viability was assessed using the colorimetric CCK-8 kit (BioSharp) to examine potential cytotoxic effects of siRNA treatment. Cells were seeded in 96-well plates and transfected with either si-DDX10 or a non-targeting control siRNA (si-NC). At 24 h and 36 h post-transfection, 10 µL of CCK-8 reagent was added to each well, followed by incubation at 37 °C for 4 h. Absorbance at 450 nm was measured using a microplate reader (Tecan). The entire experiment was independently repeated three times.

### Sequence analysis of PB1

PB1 protein sequences of H1N1, H3N2, H5N1, H7N9, and H9N2 influenza A viruses isolated from human, swine, and chicken were obtained from GISAID and the NCBI Influenza Virus Database (accessed up to 1 January 2026). Sequences were aligned using MAFFT, and the frequencies of residues at positions 298, 336, and 364 were calculated and plotted using GraphPad Prism 9.

### Statistical analysis

All quantitative data are presented as the mean ± standard deviation (s.d.) from at least three biologically independent experiments. Statistical significance was assessed using GraphPad Prism 9 software. Comparisons between two groups were performed using an unpaired, two-tailed Student’s *t*-test. For comparisons involving more than two groups or multiple variables, one-way or two-way analysis of variance (ANOVA) was applied, followed by Dunnett’s post hoc test for comparisons against a single control group, as indicated in the respective figure legends. A *P*-value of less than 0.05 was considered statistically significant (*P < 0.05; **P < 0.01; ***P < 0.001; ****P < 0.0001).

## Data Availability

The full genomes of the RB144/H1N1 swine influenza viruses first reported in this study have been deposited in GISAID under accession number EPI_ISL_20494360. Sequences for other strains (PR8/H1N1, Cal04/H1N1, HB20G/H9N2, HBJX52/H9N2, and BJ16/H9N2) are also available in GISAID, while the H9N2 strains were previously published (38).

## SUPPLEMENTAL MATERIAL

Supplemental material jvi.00815-26-s0001.docx Fig. S1 to S7; Tables S1 and S2.

ASM does not own the copyrights to Supplemental Material that may be linked to, or accessed through, an article. The authors have granted ASM a non-exclusive, world-wide license to publish the Supplemental Material files. Please contact the corresponding author directly for reuse.
