# Supplementary material for: Interaction between DEAD-box RNA helicase 10 and influenza PB1 polymerase selectively regulates influenza A virus replication
Source: J Virol. 2026 Sep 22;100(10):e00815-26. doi: 10.1128/jvi.00815-26 (PMC13712855; doi:10.1128/jvi.00815-26)
Supplement: Supplementary file 1 [file jvi.00815-26-s0001.docx]

**Interaction between DEAD-box RNA Helicase 10 and Influenza PB1 Polymerase Selectively Regulates Influenza A Virus Replication**

Rizwan Ullah^1,2,3#^, Wei Chen^1,2,3#^, Lingkai Zhang^1,2,3^, Chaoxuan Guo^1,2,3^, Bin Ren^1,2,3^, Xinyi Pei^1,2,3^, Dong Yang^1,2,3^, Qingshuai Sun^1,2,3^, Xinyu Gao^1,2,3^, Hongbo Zhou^1,3^, Liurong Fang ^1,3,4^, Wen Su ^1,2,3,5*^

^1^ National Key Laboratory of Agricultural Microbiology, College of Veterinary Medicine, Huazhong Agricultural University, Wuhan, 430070, Hubei, China

^2^ Hubei Hongshan Laboratory, Wuhan 430070, China

^3^ Frontiers Science Center for Animal Breeding and Sustainable Production, Wuhan, Hubei, 430070, China.

^4^ The Cooperative Innovation Center for Sustainable Pig Production, Huazhong Agricultural University, Wuhan, 430070, Hubei, China

^5^Shennongjia Science and Technology Innovation Center, Shennongjia Multidisciplinary Observation and Research Station of the Ministry of Agriculture and Rural Affairs, Huazhong Agricultural University, Wuhan, 430070, Hubei, China.

^#^ These authors contributed equally to this work. Author order was determined by seniority.

* vetsu@mail.hzau.edu.cn

**Supporting Information**

**FIG S1 Phylogenetic analysis of human DEAD-box RNA helicase (DDX) family members used in this study.** A phylogenetic tree was constructed based on the amino acid sequences of the DDX family proteins, using the neighbor-joining method (1,000 bootstrap replicates) in MEGA11 software. Protein names highlighted in red represent the proteins analyzed in this study. Numbers on the branches indicate bootstrap support values (%) based on 1000 replicates.

**FIG S2 Knockdown of DDX10 does not impair cell viability.** **HEK293T cells were transfected with si‑DDX10 or a non‑targeting control siRNA (si‑NC). Cell viability was measured at 24 and 36 hours post‑transfection using a CCK‑8 assay. Data represent mean absorbance ± s.d. (n=3 technical replicates) and demonstrate that DDX10 knockdown did not significantly affect cell viability under the experimental conditions.**

**FIG S3** **DDX10 overexpression does not alter BJ16/H9N2 viral protein expression. HEK293T cells were transfected with the BJ16/H9N2 mini-genome system components along with over expressing DDX10 or empty vector. Western blot analysis confirms that DDX10 overexpression does not affect the expression levels of the co-transfected viral proteins.**

**FIG S4 Influenza PB1 and NP doesn’t interact DDX24 and DDX43, whereas DDX10 interacts in an RNA‑independent manner**. (**A**) Co‑immunoprecipitation (Co‑IP) of DDX24 and DDX43 with PB1 and NP. HEK293T cells were co‑transfected with HA‑DDX24 or DDX43 and Flag‑tagged PB1or NP from Cal04/H1N1 (A). Cell lysates were subjected to anti‑HA IP followed by Western blotting with the indicated antibodies. (**B**) Lysates from cells expressing HA‑DDX10 with Flag‑PB1 or Flag‑NP, both with and without the complete polymerase complex (PB2+PA) (Cal04/H1N1) and Poll, were treated with RNase A before anti‑HA immunoprecipitation and Western blotting.

**FIG S5 DDX10 does not affect NP oligomerization. HEK293T cells were co‑transfected with plasmids expressing HA‑NP, Flag‑NP, and increasing amounts of Flag‑DDX10 (0 or 1.0 µg). Cell lysates were harvested at 36 h post‑transfection and subjected to co‑immunoprecipitation (Co‑IP) using an anti‑HA antibody, followed by Western blot analysis. The ratio of co‑precipitated Flag‑NP to HA‑NP was quantified using image j (Fiji).**

**FIG S6 Location of** **residues 298, 336 and 364 on the surface of the PB1 catalytic domain. The three-dimensional structure of viral polymerase (PDB ID: 7NHX, containing the PB1(red), PB2 (gray) and PA (blue)) is shown in cartoon representation (left panel), with the residues** **298, 336 and 364 highlighted as green. A close-up view of the PB1 catalytic domain illustrates the surface-exposed positions of residues I298, I336 and I364. The structure was visualized using PyMOL (version 3.1).**

**FIG S7 DDX10 expression is higher in A549 cells than in primary airway epithelial cells, limiting the predicted risk of H9N2 PB1 mutants. (A-B)** A549 cells, Calu‑3 and NHBE cells grown were evaluated via qPCR (A) and western blotting to measure the DDX10 expression levels. Statistical significance was determined by unpaired two‑tailed Student’s t‑test. P‑values less than 0.05 are marked, with significance levels indicated as ***P < 0.01 and ****P < 0.0001.

**Table S1 Amino acid sequence differences between Cal04/H1N1 and BJ16/H9N2 PB1 proteins.**

| Residue position (PB1) | BJ16/H9N2 | Cal04/H1N1 |
| --- | --- | --- |
| 12 | V | I |
| 14 | V | A |
| 48 | K | Q |
| 56 | A | T |
| 57 | M | T |
| 113 | I | V |
| 172 | D | E |
| 175 | D | N |
| 179 | M | I |
| 216 | S | G |
| 257 | A | T |
| 298 | L | I |
| 336 | V | I |
| 339 | I | M |
| 361 | S | R |
| 364 | L | I |
| 368 | V | I |
| 375 | N | S |
| 383 | K | E |
| 386 | R | K |
| 387 | E | K |
| 435 | T | I |
| 486 | R | K |
| 517 | I | V |
| 573 | A | S |
| 581 | E | D |
| 584 | R | Q |
| 587 | A | V |
| 618 | E | D |
| 621 | Q | R |
| 628 | M | L |
| 648 | S | A |
| 728 | I | V |
| 741 | A | S |

**Table S2 Oligonucleotide primers used in this study.**

| Primers Nucleotide sequence (5´ ̶ 3´) | |
| --- | --- |
| hDDX10-F  hDDX10-R  Cal04-PB2-F  Cal04-PB2-R  Cal04-PB1-F  Cal04-PB1-R  Cal04-PA-F  Cal04-PA-R  Cal04-NP-F  Cal04-NP-R  chiPB1-NT-F  chiPB1-NT-R  chiPB1-CD-F  chiPB1-CD-R  chiPB1-CT-F  chiPB1-CT-R  BJ16-PB1- L298I –F  BJ16-PB1- L298I –R  BJ16-PB1- V336I –F  BJ16-PB1- V336I –R  BJ16-PB1- L336I –F  BJ16-PB1- L336I –R  q-hDDX10-F  q-hDDX10-R  q-GAPDH-F  q-GAPDH-R | CCATCGATATGGGCAAAACGGCCAACTC  CCGCTCGAGTTAGCTTTGACTTCTTAGCAGATGT  CCATCGATATGGAGAGAATAAAAGAACTGAGAGA  CCGCTCGAGCTAATTGATGGCCATCCGAA  CCATCGATATGGATGTCAATCCGACTCT  CCGCTCGAGTTATTTTTGCCGTCTGAGTTCTTCA  CCATCGATATGGAAGACTTTGTGCGACA  CCGCTCGAGCTACTTCAGTGCATGTGTGAGG  CCATCGATATGGCGTCTCAAGGCACCAA  CCGCTCGAGTCAACTGTCATACTCCTCTGCA  CCATCGATATGGATGTCAATCCGACTCT  CCGCTCGAG TTAAACATTTGCCAGTTTGGCCT  CCATCGATATGGTGAGAAAGATGATGACTAATTCACA  CCGCTCGAGTTAATAGGACTTCTTTTTGCTCA  CCATCGATATGATAAATAAGACAGGGACATTTGA  CCGCTCGAG TTATTTTTGCCGTCTGAGTTCTTCA  TTCACAAGATACAGAGATCTCCTTCACAATTACTG  CAGTAATTGTGAAGGAGATCTCTGTATCTTGTGAA  AGAATGGTTTAGAAATATCTTAAGCATTGCTCCTA  TAGGAGCAATGCTTAAGATATTTCTAAACCATTCT  AAGTAAGAGTATGAAGATACGGACACAAGTACCAG  CTGGTACTTGTGTCCGTATCTTCATACTCTTACTT  ACCGTTTGGTGACTGAGATACA  TGAAGTCCATTGCAGACGATAT  GCTAAGGCTGTGGGCAAGG  GGAGGAGTGGGTGTCGCTG |
